# Primary Aldosteronism and Ischemic Heart Disease

**DOI:** 10.3389/fcvm.2022.882330

**Published:** 2022-05-23

**Authors:** Shivaraj Patil, Chaitanya Rojulpote, Aman Amanullah

**Affiliations:** ^1^Department of Cardiology, Einstein Medical Center, Philadelphia, PA, United States; ^2^Department of Medicine, The Wright Center for Graduate Medical Education, Scranton, PA, United States

**Keywords:** primary hyperaldosteronism (PA), ischemic heart disease, atherosclerosis, secondary hypertension, coronary artery disease

## Abstract

Cardiovascular disease, in particular ischemic heart disease is a major cause of morbidity and mortality worldwide. Primary aldosteronism is the leading cause of secondary hypertension, yet commonly under diagnosed, and represents a major preventable risk factor. In contrast to historical teaching, recent studies have shown that excess aldosterone production is associated with increased burden of ischemic heart disease disproportionate to the effects caused by hypertension alone. Aldosterone through its genomic and non-genomic actions exerts various detrimental cardiovascular changes contributing to this elevated risk. Recognition of primary hyperaldosteronism and understanding the distinctive pathophysiology of ischemic heart disease in primary aldosteronism is crucial to develop strategies to improve outcomes.

## Introduction

Cardiovascular diseases (CVDs), consisting of ischemic heart disease, heart failure, peripheral arterial disease, and several other cardiac and vascular conditions, constitute the leading cause of global mortality and are a major contributor to reduced quality of life (1). Amongst CVDs, ischemic heart disease (IHD) is the main global cause of death, accounting for more than 9 million deaths in 2016 according to the World Health Organization (WHO) estimates. Although IHD rates are decreasing globally, risk factor prevalence is rising (2). Hypertension is a major modifiable risk factor for ischemic heart disease with nearly 1.39 billion adults affected worldwide in 2010 (3). It is estimated that risk of a fatal coronary event doubles with an increase in systolic blood pressure of 20 mm Hg or each 10-mm Hg increase in diastolic blood pressure (4). Aldosterone, a steroid hormone secreted by the adrenal gland, is an important regulator of blood pressure and primary hypersecretion of this hormone leads to dysregulation of homeostatic control mechanisms. Moreover, normotensive individuals with higher plasma aldosterone levels within physiological range are at an increased risk of subsequent rise in blood pressure and development of incident hypertension (5). The effect of excess production of aldosterone has traditionally been conceptualized as a result of its action on renal collecting duct principal cells, via mineralocorticoid receptors (MR), to induce sodium and water reabsorption and potassium excretion clinically culminating in hypertension associated with low or low-normal serum potassium levels. Historically, primary aldosteronism was considered an uncommon disease, however, with advances in diagnostic technology, primary aldosteronism (PA) is now identified as the most common endocrinological cause of secondary hypertension, with aldosterone producing adenoma (APA) and bilateral adrenal hyperplasia (BAH, idiopathic hyperaldosteronism) accounting for vast majority of cases (6). Recent evidence has linked primary aldosteronism with increased cardiovascular morbidity and mortality, out of proportion to the adverse effects caused solely by elevated blood pressure, underscoring its pleiotropic effect and multifaceted role in cardiovascular pathophysiology (7–9). In this review, we aim to focus on the prevalence and pathophysiologic mechanisms of IHD in primary aldosteronism.

## Prevalence of Ischemic Heart Disease in Primary Aldosteronism

The true prevalence of PA is underestimated, with current studies reporting a prevalence of 3–13% in primary care setting and up to 30% in referral centers (6). The prevalence of IHD and MI in patients with primary aldosteronism is variable depending on the study design and diagnostic criteria ranging from 1.7 to 20% and 0.9 to 4.4%, respectively. The pooled prevalence of IHD and MI in PA were estimated to be 3.4 and 1.7% respectively (Table 1). Various epidemiological studies clearly demonstrate that individuals with primary aldosteronism experience higher burden of IHD compared to individuals with essential hypertension with comparable demographic and cardiovascular risk factor profile (8, 10, 11). Individuals with PA presenting with unilateral subtype, or plasma aldosterone concentration ≥125 pg/ml are at a greater risk of CVD (10). It has also been observed that hypokalemic variant of primary aldosteronism is associated with excessive burden of ischemic heart disease compared to normokalaemia variant, possibly due to effects of higher concentration of aldosterone exposure (12). Additionally, patients with primary hyperaldosteronism are more likely to have experienced an ischemic cardiovascular complication (non-fatal myocardial infarction or angina) at the time of diagnosis of PA than otherwise similar patients with essential hypertension (11). These data should draw the clinician's attention to broaden the scope to suspect PA, especially when severity of IHD or CVD morbidity is considered out of proportion to that effectuated by essential hypertension.

**Table 1:** Prevalence of ischemic heart disease in primary aldosteronism.

**Study**	**Age (years)**	**Gender (men %)**	**SBP (mmHg)**	**DBP (mmHg)**	**IHD (%)**	**MI (%)**
Ohno et al. (10) (Japan) (*N* = 2,582)	53.2 ± 11.3	47.1	141.4 ± 18.2	86.5 ± 12.8	2.1	0.9
Mulatero et al. (49) (Italy) (*N* = 270)	44 ± 8.5	59.6	155 ± 21	96 ± 12	2.6	4.0
Savard et al. (11) (France) (*N* = 459)	51.1 ± 10.2	67	151 ± 24.4	87.7 ± 13.1	5.7	4.4
Reincke et al. (50) (Germany) (*N* = 300)	50.0	61	168 ± 25	99 ± 16	4	-
Milliez et al. (51) (France) (*N* = 124)	52 ± 10	67	176 ± 23	107 ± 14	-	4
Choi et al. (52) (South Korea) (*N* = 85)	46.1 ± 10.3	43.5	173.8 ± 33.6	106.1 ± 19.2	20	-
Nishimura et al. (53) (Japan) **(***N* = 58)	45 ± 9	53.4	166 ± 30	96.5 ± 18	1.7	-
Catena et al. (8) (Italy) **(***N* = 54)	53 ± 12	70.4	167 ± 16	103 ± 9	20	-
Pooled prevalence IHD/MI (*N* = 3,808/3,435)					3.4	1.7

*SBP, systolic blood pressure; DBP, diastolic blood pressure; IHD, ischemic heart disease; MI, myocardial infarction*.

## Pathophysiology of Ischemic Heart Disease in Primary Aldosteronism

**Figure 1:**
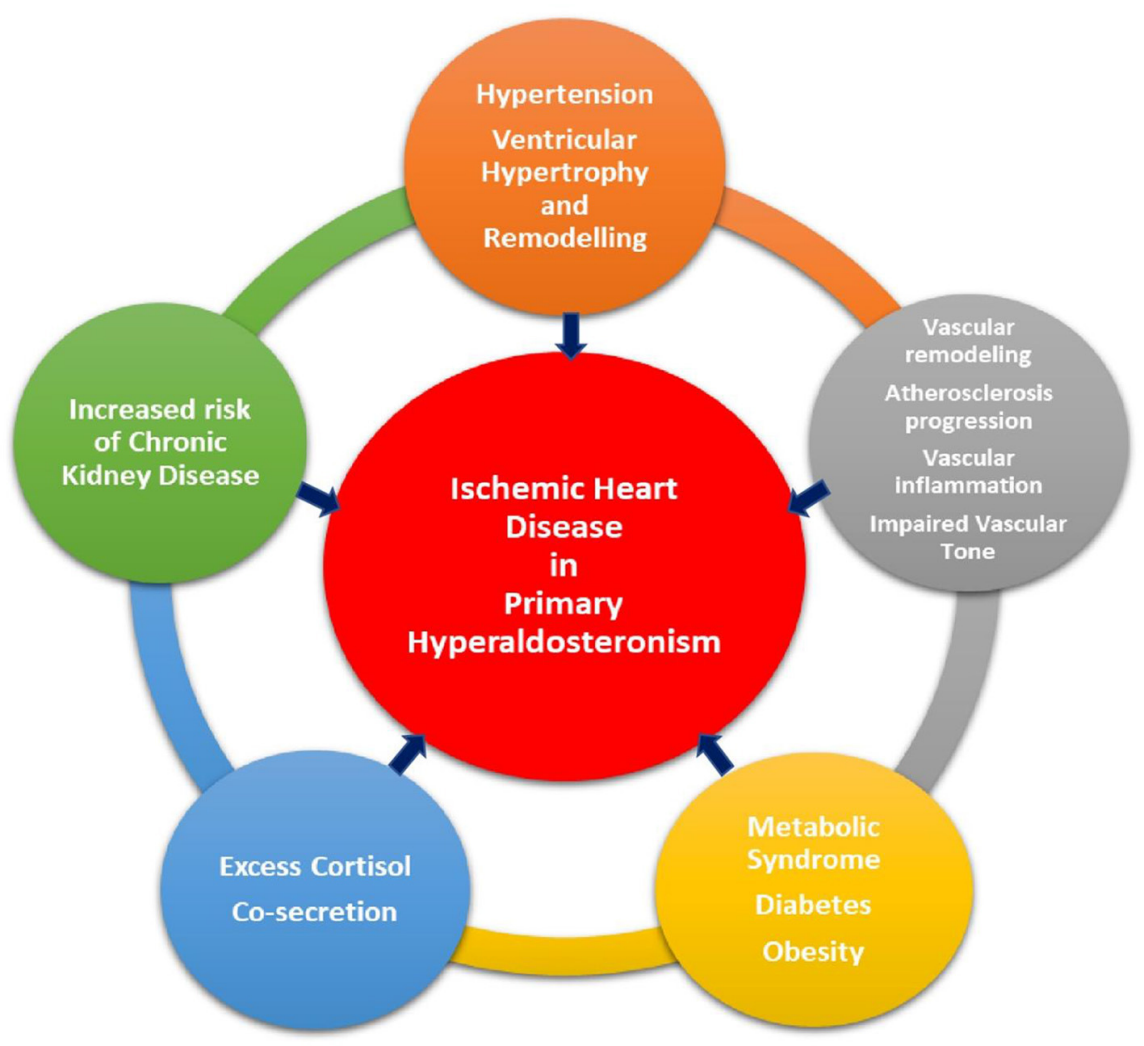
Pathways through which primary aldosteronism plays a role in the development of ischemic heart disease.

IHD occurs due to an imbalance between myocardial blood supply and myocardial demand, either at rest or exertion. Atherosclerosis is the major pathophysiological process involved in development of IHD. IHD can be silent, termed as silent myocardial ischemia, or manifest clinically either as acute coronary syndrome, secondary to plaque rupture and coronary thrombosis, or as stable angina, due to fixed narrowing of the coronary arteries from plaque buildup in the vessel wall and luminal narrowing. Conventional risk factors for IHD include increased age, hypertension, diabetes mellitus, smoking, hyperlipidemia, and family history of IHD. Excess aldosterone not only adversely affects the vasculature and cardiac muscle, but also influences cardiovascular risk factors *via* various biochemical pathways, uniquely contributing to the development of IHD (Figure 1).

### Effect of Hyperaldosteronism on Blood Pressure and Left Ventricle

It is well known that elevated aldosterone levels raise blood pressure via its genomic action mediated through mineralocorticoid receptor (MR) to absorb sodium and water in the renal collecting ducts causing volume expansion. Additionally, hyperaldosteronism increases blood pressure mediated *via* its harmful effect on vascular remodeling. Hypertension due to PA is often undiagnosed and chronic exposure to elevated blood pressure, results in compensatory left ventricular (LV) hypertrophy. In addition, long term exposure of cardiac myocyte to elevated aldosterone levels leads to myocyte hypertrophy by increasing myocardial expression of cardiotrophin-1 (CT-1), a cytokine that induces expression of myosin light chain and skeletal α-actin and enhances myosin light chain phosphorylation (13). In addition, exposure to aldosterone causes increased mRNA levels of α- and β-myosin heavy chain via activation of mineralocorticoid receptors (MRs), extracellular signal-regulated kinase (ERK), c-Jun N-terminal kinase and protein kinase C-α (14). Remodeling of left ventricle in PA not only occurs via cardiac myocyte hypertrophy but also simultaneously via cardiac fibrosis through chronic inflammation, and dysregulation of extra cellular matrix metabolism (15). This results in stiffening of the LV with subsequent elevation in LV end-diastolic pressure (LVEDP). High LVEDP compromises diastolic coronary blood flow (CBF) by decreasing the coronary perfusion pressure (CPP) leading to decreased myocardial oxygen supply (16, 17). A combination of increased LV mass and diminished diastolic CBF causes supply-demand mismatch resulting in myocardial ischemia.

### Effect of Hyperaldosteronism on Vasculature

Elevated serum aldosterone levels in PA patients lead to detrimental effects on the endothelium via genomic and nongenomic mechanisms via mineralocorticoid receptor-dependent and independent manners. Excess generation of reactive oxygen species (ROS) via increased NADPH oxidase production, decreased endothelial expression of G6PD, and mitochondrial ROS generation in the electron transport chain and release result in oxidative stress and amplify vascular inflammation. These processes are thought to be mediated via MR-independent (via extracellular-signal-regulated kinase (ERK) 1/2, c-Jun N-terminal kinase (JNK) and GPER pathways) and MR-dependent pathways. Aldosterone also increases the expressions of adhesion molecules, namely ICAM1 and VCAM-1, and inflammatory markers, such as COX-2 and MCP-1 in the endothelium, which induces monocytes and macrophage infiltration. The infiltrated monocyte-derived macrophages which are rich in NADPH oxidase further augment the generation of ROS and worsen vascular inflammation. The infiltrated macrophages ingest oxidized low-density lipoproteins and become foam cells, which potentiate the formation of atherosclerotic plaque. Aldosterone promotes inflammatory plaque formation via placental growth factor (PlGF) which binds to VEGF type 1 receptors on endothelial and inflammatory cells, and further promotes vascular smooth cell proliferation and monocyte chemotaxis, which are fundamental processes of atherosclerosis. Aldosterone also increases the formation of vasoconstrictors and decreases production and bioavailability of nitric oxide causing impairment of vascular relaxation. Aldosterone via MR-dependent pathways in the endothelium and vascular smooth muscle cells induces vascular fibrosis contributing to vascular stiffness and remodeling (18, 19).

### Effect of Hyperaldosteronism on Obesity, Diabetes, and Metabolic Syndrome

Diabetes is a well-established risk factor for IHD and is considered a coronary artery disease equivalent (20). PA is linked to increased risk of diabetes and metabolic syndrome. Clustering of hypertension, abdominal obesity, dyslipidemia and impaired glucose metabolism, is more commonly encountered in PA patients than individuals with essential hypertension (21, 22). Hyperaldosteronism is thought to cause increase in fat mass through mineralocorticoid receptor activation in adipocytes, which in turn induce excess aldosterone production through the actions of adipocytokines (CTRP1, leptin, and resistin) and activation of the sympathetic nervous system, which turns on the renin-angiotensin-aldosterone system, thereby creating a vicious cycle (23, 24). Deranged glucose metabolism occurs through aldosterone mediated impaired insulin sensitivity in skeletal muscle and adipose tissue *via* the MR receptor, and impaired insulin secretion, albeit the underlying mechanisms leading to decreased insulin secretion are poorly understood (25). In addition, blockade of MR has shown to improve coronary flow reserve on cardiac PET scan among individuals with type 2 diabetes without clinical evidence of ischemic heart disease, suggesting that excess MR activation in diabetes contributes to coronary microvascular dysfunction (26).

### Effect of Hyperaldosteronism on Kidneys

Primary aldosteronism causes renal dysfunction, independent of blood pressure, by inducing renal fibrosis, vascular remodeling, and podocyte injury *via* MR stimulation from excess aldosterone production (27). Chronic kidney disease is an independent risk factor for development of ischemic heart disease (28). Renal dysfunction increases oxidative stress, imparts endothelial dysfunction, and promotes systemic inflammation which accelerates atherosclerosis.

## Role of Hypercortisolism in Primary Hyperaldosteronism

Individuals with PA frequently have excess cortisol co-secretion, which can further worsen cardio-metabolic risk through their synergistic effects (29, 30). Cortisol is normally converted to an inactive metabolite, cortisone, by the action of 11β-hydroxysteroid dehydrogenase type 2(11β-HSD2). However, in hypercortisolism the activity of this enzyme is decreased, albeit due to unclear reasons (31). Loss of 11β-HSD2 has been shown to promote atherogenesis *via* activation of MR stimulating pro-inflammatory processes in the endothelium of knock-out murine models (32). Likewise, individuals with hypercortisolism demonstrate an increased burden of coronary calcification and noncalcified coronary plaque. Additionally, hypercortisolism promotes a prothrombotic state (33). This phenotype of PA and glucocorticoid co-secretion underscores the importance of additional screening for hypercortisolism due to therapeutic and prognostic implications (34).

## Role of Aldosterone Excess Post-acute Myocardial Infarction

After acute myocardial infarction (MI) circulating levels of serum aldosterone are elevated as a consequence of neurohormonal activation (35). Hyperaldosteronism after acute MI effectuates a myriad of maladaptive changes in the post-MI heart which increase morbidity and mortality (36, 37). Post-MI hyperaldosteronism contributes to ventricular remodeling that involves both infarcted and non-infarcted zones, which at a cellular level occurs through myocyte apoptosis, myocyte hypertrophy, macrophage/monocyte infiltration, and collagen deposition *via* fibroblast activation and proliferation. Excess aldosterone also induces endothelial dysfunction by reducing nitric oxide generation and increasing formation of reactive oxygen species (38). Moreover, elevated aldosterone leads to electrical remodeling, lengthened action potential duration, increase in Ca^2+^ current (*I*_Ca_) and a decrease in K^+^ transient outward current (*I*_to_), even before morphological remodeling occurs, creating a pro-arrhythmogenic milieu (39).

The Eplerenone Post-Acute Myocardial Infarction Heart Failure Efficacy and Survival Study (EPHESUS) showed beneficial effect of MR antagonists when utilized in the early post-MI period, namely by decreasing the incidence of sudden cardiac death and heart failure hospitalizations (40). Current guidelines recommend treatment with MR antagonists in patients with acute MI with ejection fraction <40% and clinical heart failure or diabetes (41, 42). Given the unfavorable effects of hyperaldosteronism in the post-MI setting, and the positive impact of aldosterone antagonists among patients with post MI systolic heart failure, the role aldosterone antagonists in improving outcomes in post-MI patients without systolic heart failure has garnered incredible clinical interest. A recent pilot study showed MR antagonists when initiated prior to reperfusion in STEMI patients resulted in improvement in ventricular remodeling at the end of 3 months, however, no impact on reducing MI size was seen (43). Despite abundant preclinical and mechanistic data supporting the concept, no clinically meaningful benefit, i.e., reduction in overall or cardiovascular mortality, ventricular arrhythmia, or rehospitalization, with use of MR antagonists in early post-MI patients without evidence of heart failure has been demonstrated in major prospective randomized clinical trials (44, 45). This underscores the need for more adequately powered prospective randomized trials evaluating the safety and efficacy of MRA administration in early post-MI patients without evidence of HF.

## Effect of Primary Aldosteronism Treatment on Ischemic Heart Disease

If diagnosed, patients with PA can be offered targeted treatment, either in the form of unilateral adrenalectomy for APA, or mineralocorticoid receptor antagonists, typically used for BAH, and sometimes for APA who are unable or unwilling to undergo adrenalectomy. Despite the presence of increased cardiovascular morbidity in PA patients at the time of diagnosis, administration of appropriate treatment results in improved cardiovascular outcome, when the effects of excess aldosterone are permanently removed. Younger age and shorter duration of hypertension independently predict beneficial cardiovascular outcomes, underscoring the importance of a timely correction of this disorder (8). Surgical adrenalectomy appears to be superior in mitigating adverse cardiovascular events compared to medical therapy in unilateral PA (46). PA patients on MR antagonist therapy with unsuppressed plasma renin activity (PRA) ≥ 1 ng/ml/h, a marker of effective MR blockade, seem to have comparable cardiovascular outcomes to those with essential hypertension. In contrast, patients with suppressed PRA <1 ng/ml/h experience poorer cardiovascular outcomes despite similar blood pressure control (47). Future prospective studies are necessary to determine treatment approaches in patients with PA to optimize cardiovascular outcomes. Given the reversal of this increased cardiovascular risk through therapy, a robust effort to diagnose and effectively treat PA, undoubtedly reduces health costs and improves quality of life (48).

## Conclusion

Primary aldosteronism is common, with true prevalence expected to be higher than current estimates. Furthermore, it carries a significantly worse cardiovascular prognosis compared to individuals with essential hypertension. Early detection of this entity could not only improve outcomes for patients but also potentially be cost saving for the healthcare system.

## Author Contributions

SP and CR made substantial contribution to the article design and conception of the work, contributed to the acquisition of data, and drafting and editing of the manuscript. AA and SP made critical revisions. All authors read and approved the final manuscript.

## Funding

This work was funded by Einstein Medical Center, Philadelphia.

## Conflict of Interest

The authors declare that the research was conducted in the absence of any commercial or financial relationships that could be construed as a potential conflict of interest.

## Publisher's Note

All claims expressed in this article are solely those of the authors and do not necessarily represent those of their affiliated organizations, or those of the publisher, the editors and the reviewers. Any product that may be evaluated in this article, or claim that may be made by its manufacturer, is not guaranteed or endorsed by the publisher.
